# ﻿Confirmation of the existence of Himalayan long-eared bats, *Plecotushomochrous* (Chiroptera, Vespertilionidae), in China

**DOI:** 10.3897/zookeys.1161.99487

**Published:** 2023-05-11

**Authors:** Pengfei Luo, Xiangyang He, Yuzhi Zhang, Jianping Ye, Min Guo, Jin Deng, Chunhui Zhou, Jiang Zhou, Libiao Zhang

**Affiliations:** 1 Guangdong Key Laboratory of Animal Conservation and Resource Utilization, Guangdong Public Laboratory of Wild Animal Conservation and Utilization, Institute of Zoology, Guangdong Academy of Sciences, Guangzhou 510260, China Institute of Zoology, Guangdong Academy of Sciences Guangzhou China; 2 School of Karst Science, State Engineering Technology Institute for Karst Desertification Control, Guizhou Normal University, Guiyang 550001, China Guizhou Normal University Guiyang China; 3 Guangxi Guilin Maoershan National Nature Reserve Administration, Guilin 541000, China Guangxi Guilin Maoershan National Nature Reserve Administration Guilin China

**Keywords:** cyt *b* gene, morphology, echolocation calls

## Abstract

The existence of Himalayan long-eared bats, *Plecotushomochrous* (Chiroptera, Vespertilionidae), in China has not been previously confirmed. In this study, four bats captured with harp traps from two sites in the Maoershan National Nature Reserve in Guangxi, China were investigated. These bats have long, wide auricles, each with a prominent tragus. The length of each auricle is about the same as that of a forearm. Hairs on the ventral fur have a dark base with mixed grey and yellowish tips; those on the dorsal fur also have a dark base and are bicolored with brown tips. The thumbs are very short. A concavity is present in the front of the dorsal side of the cranium. Based on morphological characteristics and phylogeny using Cyt *b* gene sequences, these bats were identified as *P.homochrous*, thus confirming the existence of Himalayan long-eared bats in China.

## ﻿Introduction

As bats of various species of the genus *Plecotus* E. Geoffroy, 1818 are morphologically very similar (Spitzenberger et al. 2006), taxonomic classification of them is very difficult. In 1847, Hodgson described the bats that he found in Nepal as Himalayan Long-eared bats (*Plecotushomochrous* Hodgson, 1847). However, this taxon was later considered a subspecies of *P.auritus* (Linnaeus, 1758) (Ellerman and Morrison-Scott 1951; Hanák 1966; Corbet 1978; Koopman 1993; Wang 2003; Simmons 2005). Horácek et al. (2000) proposed that *P.homochrous* should be considered an independent species based on its biogeographical characteristics. Later, Spitzenberger et al. (2006) revised the taxonomic status of all species in the genus based on results of morphological and molecular analyses and classified *P.homochrous* as a distinct species.

The first evidence for the existence of *P.homochrous* in China was reported by Wang (2003) who identified the bats he found in Xinping County, Yunnan Province, China as *P.auritushomochrous*. However, this record was not acknowledged by Simmons (2005), Wilson and Mittermeier (2019), Jiang et al. (2021), and Wei et al. (2021). Therefore, the existence of *P.homochrous* in China remained uncertain, and *P.homochrous* were believed to occur only in the southern Himalayas and Southeast Asia, including northern Pakistan, northwestern India, Nepal, and Vietnam (Wilson and Mittermeier 2019; Dai et al. 2020). In this study, we confirm the existence of *P.homochrous* in China and report on their morphological characteristics, phylogenetic relationships, and echolocation call patterns.

## ﻿Materials and methods

### ﻿Sample collection

**Figure 1.**
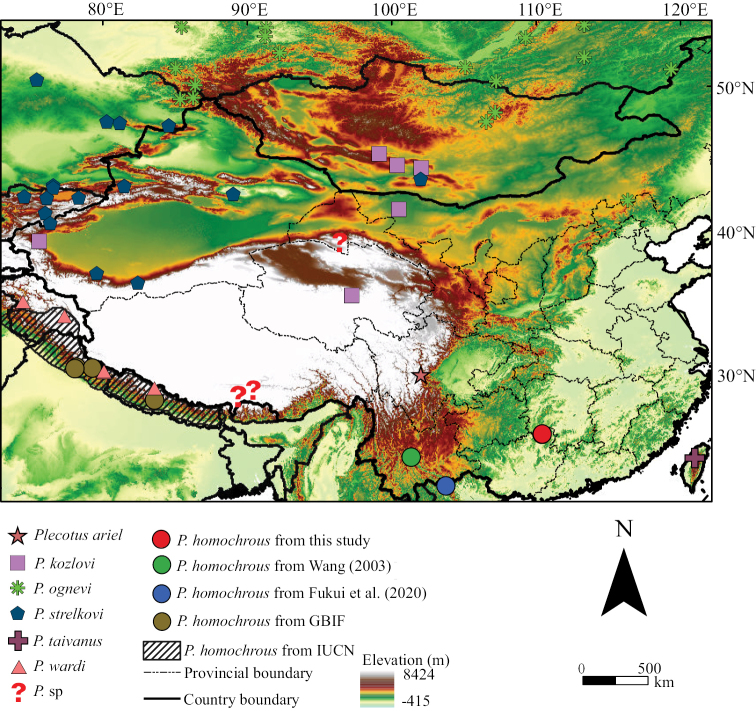
Distribution of *Plecotus* bats in China and other regions. The map shows the southernmost regions, not the entire China, where *Plecotus* bats have been found.

Bats examined in this study were captured from the Maoershan National Nature Reserve (25°48'N–25°58'N, 110°20'E–110°35'E), which covers an area of 170.09 km^2^ of mountains with varied vegetation types. Although some areas at lower elevations have been transformed into bamboo forests, most of the reserve is undisturbed with primary forests, especially at higher elevations (Huang and Jiang 2002). Four *Plecotus* bats were captured from two sites (Fig. 1; 25°26'20"N, 110°53'32"E, 2002 m a.s.l. and 25°54'42"N, 110°27'14"E, 1708 m a.s.l.) with harp traps during a bat survey along an elevational gradient in June 2022. These bat specimens, designated GD-221656, GD-221657, GD-221658, and GD-221659, were preserved in anhydrous ethanol after all examinations were completed. These specimens are stored at the Guangdong Institute of Zoology.

### ﻿Morphological measurements and recording of echolocation calls

Morphological measurements of bats were performed with electronic digital calipers according to Dai et al. (2020). Definitions of the measurements are as follows: FA, forearm length; T, tail length; HB, head and body length; Thsu, thumb length excluding claws; Thcu, thumb length including claws; Tib, tibia length; Hfsu, hindfoot length excluding claws; Hfcu, hindfoot length including claws; Trag, tragus length; E, ear length; STOTL, total length of the skull; CBL, condylobasal length; CCL, condylo-canine length; MAW, mastoid width; CM^3^L, maxillary toothrow length; CCW, width across upper canines; M^3^M^3^W, width across upper molars; CM_3_L, mandibular tooth row length; ML, mandible length; UJH, lower jaw height; BCW, braincase width; BCH, braincase height; ZYW, zygomatic width; RL, rostral length; Bulla, diameter of tympanic bulla; IOW, interorbital width. The wing shape of each bat was recorded by tracing on paper, followed by a determination of wing loading and wingspan ratio using IMAGE J according to the method of Norberg and Rayner (1987). The criteria of Bininda-Emonds and Russell (1994) and Aldridge and Rautenbach (1987) were used for classification of wingspan ratio and wing loading as follows: wingspan ratio: low, 6.1–7.3; high, ≥7.3; wing loading: very low, ≤6.45 N/m^2^; low, 6.45–10.3 N/m^2^; high, ≥10.3 N/m^2^.

Morphological measurements of six *Plecotus* species (Suppl. material 1: table S1) were used for the principal component analysis (PCA) using ‘prcomp’ function of the R package ‘stats’ (R Core Team 2021). The following 10 craniodental measurements were assessed by PCA: STOTL, CBL, MAW, CM^3^L, M^3^M^3^W, CM_3_L, BCW, BCH, Bulla, and IOW (Suppl. material 1: table S2).

Echolocation calls of four bats were recorded using a handheld ultrasound detector (UltraSoundGate 116Hm, Avisoft Bioacoustis, Germany) when they were allowed to fly in a room of 5 × 5 × 2.5 m^3^ in size. Ultrasound spectrograms were generated using the 512-point Fast Fourier Transform (FFT) algorithm with 96.87% of the frequency overlapped with a Hanning window. A total of 30 pulses were arbitrarily selected from each bat for determination of start frequency, end frequency, frequency of maximum energy, and pulse duration using the Batsound software (Pettersson Elektronik AB, Uppsala, Sweden). The values were determined based on the second (highest energy) harmonic and statistically compared with those of the study from Vietnam (Dai et al. 2020) using ‘kruskal.test’ function of the R package ‘stats’ (R Core Team 2021) as the data were non-normally distributed as determined by the Shapiro-Wilk and normal Q-Q plot.

### ﻿Phylogenetic analyses

To further identify the bats, DNA was extracted from a small piece of the wing membrane of each bat, and polymerase chain reaction was performed to amplify a portion of the mitochondrial cytochrome *b* gene (Cyt *b*) using primers Cyt *b*-F (5′-TAG AAT ATC AGC TTT GGG TG-3′) and Cyt *b*-R (5′-AAA TCA CCG TTG TAC TTC AAC-3′) (Li et al. 2006). Each PCR was conducted in a volume of 50 μl containing 8 μl of genomic DNA, 2 μl each of primer F and R (10 mM each), 13 μl of water, and 25 μl of HiFi DNA polymerase master mix. PCR conditions were as follows: 5 min at 94 °C, followed by 10 cycles of 60 s at 94 °C, 30 s at 46 °C, and 62 s at 72 °C; 25 cycles of 60 s at 94 °C, 40 s at 50 °C (+0.3 °C/cycle), and 60 s at 72 °C; 35 cycles of 60 s at 94 °C, 40 s at 54 °C, 60 s at 72 °C, and final elongation for 10 min at 72 °C.

The obtained sequences were deposited in GenBank under the following accession numbers: OP425735 (GD-221657), OP425736 (GD-221659), and OP425737 (GD-221656). No sequences were obtained from bat GD-221658 because of a failure in DNA isolation. The sequences were aligned with those of 30 Cyt *b* genes (Table 4) from GenBank for phylogenetic analysis using MAFFT software (Katoh and Standley 2013). Selection of the best-fit nucleotide substitution model was performed by MODELFINDER (Kalyaanamoorthy et al. 2017), and the phylogenetic tree was constructed using the maximum-likelihood (ML) method in IQ-TREE with 5,000 ultrafast bootstraps (Nguyen et al. 2015).

## ﻿Results

### ﻿Morphological characteristics

In PCA, the percentages of explained variance of the first two principal components (PC1 and PC2) were 65.8% and 12.3%, respectively, with a cumulative percentage of 78.1% (Suppl. material 1: table S2). PC1 results were derived from all measurements except those of tympanic bullae (Bulla) and interorbital width (IOW), whereas results of PC2 were from analysis of Bulla and IOW (Table 1). PCA plots revealed that the four investigated bats were clustered with *P.homochrous* from Vietnam but were widely separated from other bats including *P.ariel* (Thomas, 1911), *P.kozlovi* (Bobrinski, 1926), *P.ognevi* (Kishida, 1927), *P.strelkovi* (Spitzenberger, 2006), and *P.wardi* (Thomas, 1911). This result suggests that these four bats are *P.homochrous*.

**Table 1.** External and cranial measurements (in mm) of *Plecotushomochrous* bats.

	**Guangxi, China**	**Lao Cai, Vietnam**
	**This study**	**Dai et al. 2020**
Body sites measured	GD-221656(♂) / GD-221657(♂) / GD-221658(♂) / GD-221659(♀)	IEBR-M-5469(♀) / IEBR-M-5472(♀) / IEBR-M-5483(♂) / HNHM202011(♀)
FA	37.30 / 37.28 / 37.36 / 38.49	38.09 / 37.36 / 37.75 / 37.58
T	39.63 / 42.01 / 44.15 / 43.12	49.00 / 45.00 / 44.00 / 47.00
HB	50.49 / 50.75 / 46.92 / 45.77	45.00 / 42.50 / 37.50 / 42.50
Thsu	3.82 / 3.28 / 3.84 / 4.46	5.34 / 4.78 / 5.11 / 4.89
Thcu	4.86 / 4.14 / 4.73 / 5.61	6.22 / 5.89 / 5.71 / 5.64
Tib	17.84 / 17.02 / 17.07 / 18.49	17.40 / 18.00 / 16.80 / 17.00
Hfsu	7.96 / 8.18 / 8.56 / 8.38	7.98 / 7.64 / 7.96 / 7.99
Hfcu	8.68 / 8.70 / 9.03 / 9.11	9.18 / 8.32 / 8.85 / 8.86
Trag	17.29 / 14.54 / 15.76 / 15.88	18.00 / 17.00 / 18.00 / 18.00
E	36.43 / 38.85 / 38.12 / 39.12	38.00 / 39.00 / 37.00 / 39.50
STOTL	16.02 / 16.34 / 16.43 / 16.37	16.03 / 16.00 / 15.35 / 15.61
CBL	14.92 / 14.94 / 15.21 / 14.98	14.79 / 14.88 / 14.28 / 14.45
CCL	14.13 / 14.20 / 14.45 / 14.23	14.38 / 14.33 / 13.74 / 14.05
MAW	8.70 / 8.81 / 8.69 / 8.79	8.95 / 8.94 / 8.41 / 8.70
CM^3^L	5.12 / 5.13 / 5.19 / 5.08	5.33 / 5.02 / 5.05 / 5.23
CCW	3.51 / 3.35 / 3.27 / 3.53	3.65 / 3.59 / 3.56 / 3.52
M^3^-M^3^	5.77 / 5.72 / 5.71 / 5.69	6.00 / 5.50 / 5.56 / 5.63
CM_3_L	5.74 / 5.60 / 5.75 / 5.65	5.70 / 6.00 / 5.27 / 5.27
ML	9.67 / 9.69 / 9.95 / 9.71	10.38 / 10.54 / 9.90 / 9.96
UJH	2.78 / 2.88 / 2.90 / 2.91	3.01 / 3.16 / 2.86 / 3.01
BCW	7.30 / 7.34 / 7.17 / 7.30	7.76 / 7.53 / 7.75 / 7.83
BCH	5.67 / 5.29 / 5.25 / 5.07	5.89 / 5.99 / 5.83 / 5.86
ZYW	8.13 / 8.27 / — / 8.22	8.32 / — / — / 8.12
RL	3.32 / 3.21 / 3.47 / 3.25	4.02 / 3.97 / 3.64 / 4.05
Bulla	4.32 / 4.22 / 4.20 / 4.43	4.41 / 4.25 / 4.18 / 4.47
IOW	3.68 / 3.43 / 3.31 / 3.56	3.63 / 3.76 / 3.63 / 3.69

**Figure 2.**
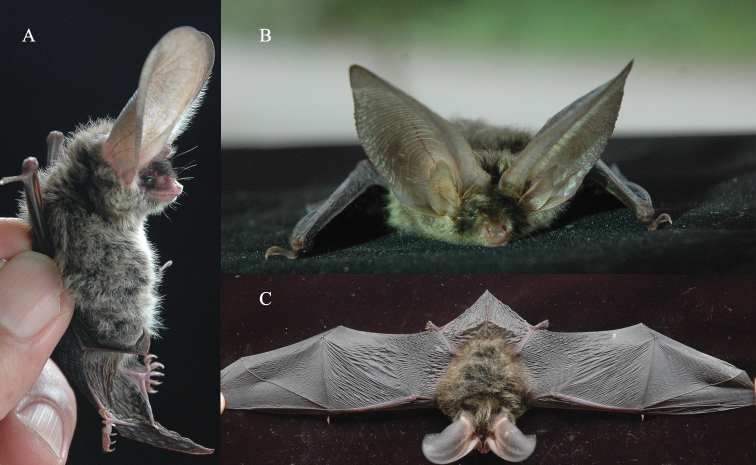
Pictures of *Plecotushomochrous* (GD-221656) examined in this study **A** left side **B** face **C** dorsal side.

**Figure 3.**
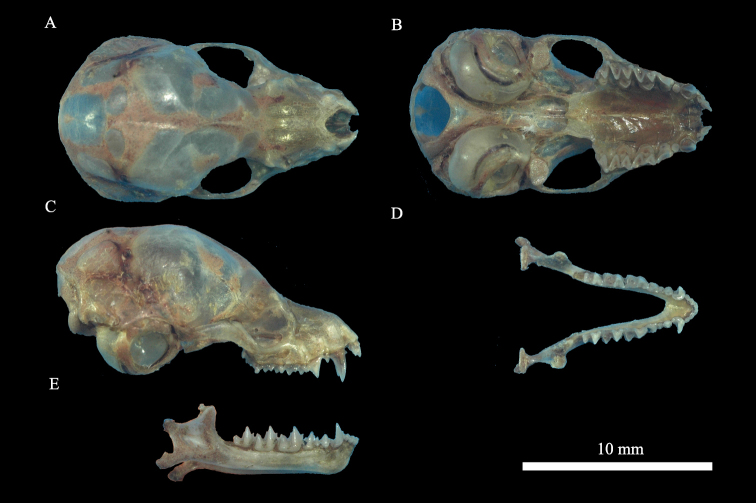
Cranial morphology of *Plecotushomochrous* (GD-221656) **A** cranium in dorsal view **B** cranium in ventral view **C** cranium in left side view **D** mandible in dorsal view **E** mandible left side view.

Morphologically, the bats have long, wide auricles, each with a prominent tragus (Fig. 3B). The length of each auricle is about the same as that of a forearm (Table 1). The bases of the two ears intersect at the forehead (Fig. 2A). Hairs on the ventral fur have a dark base with mixed grey and yellowish tips; those on the dorsal fur also have a dark base and are bicolored with brown tips (Fig. 2A, C). The facial fur is dark, and the skin is pink (Fig. 2B). The thumbs are very short (Fig. 2C). The wing membrane is attached to the base of toes, and there is a small, triangular protrusion at the base of the tail membrane near the heel (keeled calcar) (Fig. 2A). The dental formula is I 2/3, C 1/1, P 2/3, and M3/3. The first upper incisor is double pointed and higher than the second upper incisor. The second upper premolar is absent (Fig. 3B). The cranium is 16.02–16.43 mm in length and 8.13–8.27 mm in zygomatic width (Table 1), with a slight sagittal crest, which is the smallest among all *Plecotus* species. The bullae are medium-sized (diameter 4.20–4.43 mm). A concavity is present in the front of the dorsal side of the cranium (Fig. 3C). The orbital ridge is in the anterior part of the eye socket (Fig. 3A). All these morphological characteristics are identical to those of *P.homochrous* from Vietnam (Dai et al. 2020).

### ﻿Echolocation calls and wing characteristics

**Figure 4.**
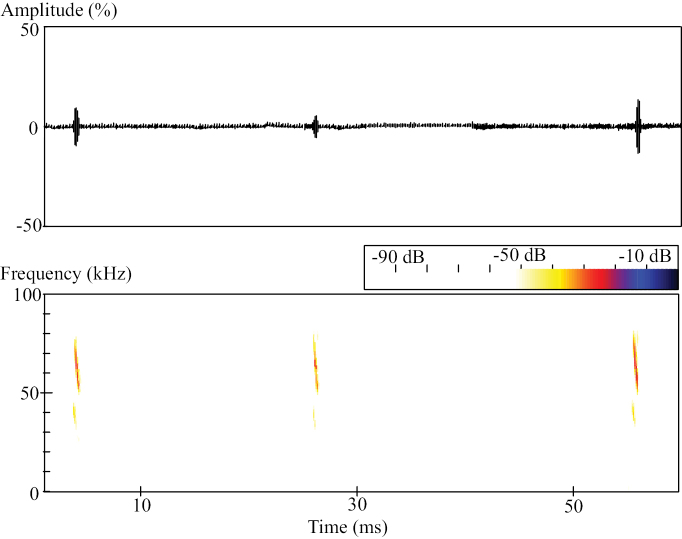
Amplitude and spectrogram of echolocation calls of bats examined in this study.

Echolocation calls of the four bats are of frequency-modulation (FM) with multiple harmonics. The maximum energy of calls is mostly in the second harmonic (Fig. 4). Sound parameters of echolocation calls vary among the four individuals. Start frequency, end frequency, frequency of maximum energy, and pulse durations are 74.0 ± 2.8 kHz, 52.2 ± 1.9 kHz, 58.7 ± 0.6 kHz, and 1.5 ± 0.2 ms (Mean ± SD), respectively. There is no significant difference in start frequency, end frequency, and frequency of maximum energy between the *P.homochrous* bats from Vietnam and the four bats examined in this study (*P* values 0.16, 0.53, and 0.26) (Table 2). However, there is a significant difference in pulse duration (*P* value 0.01). The four bats also have a very low wing loading (5.68 ± 0.29 N/m^2^) and a low wingspan ratio (6.82 ± 0.70) (Table 3), indicative of slow and flexible flights.

**Table 2.** Sound parameters of *Plecotushomochrous* echolocation calls.

Specimens	Country	Start frequency (kHz)	End frequency (kHz)	Frequency of maximum energy (kHz)	Duration (ms)
GD-221656	China	70.8	53.6	57.8	1.4
GD-221657	China	72.9	53.6	59.1	1.4
GD-221658	China	73.8	52.6	59.2	1.9
GD-221659	China	78.5	49.0	58.5	1.3
Mean ± SD		74.0 ± 2.8	52.2 ± 1.9	58.7 ± 0.6	1.5 ± 0.2
IEBR-M-5469	Vietnam	69.6	51.6	59.3	1.1
IEBR-M-5483	Vietnam	71.8	53.3	62.6	1.1
HNHM202011	Vietnam	71.2	55.3	59.3	1.1
Mean ± SD		70.9 ± 0.9	53.4 ± 15	60.4 ± 1.6	1.1 ± 0.0
Kruskal–Wallis test		ns	ns	ns	*P* = 0.01

**Table 3.** Wing characteristics of *Plecotushomochrous* from China.

Specimens	Wingspan ratio (N/m^2^)	Wingload
GD-221656	8.01	5.42
GD-221657	6.44	5.41
GD-221658	6.57	6.09
GD-221659	6.26	5.81
Mean ± SD	6.82 ± 0.70	5.68 ± 0.29

### ﻿Phylogenetic analysis

**Figure 5.**
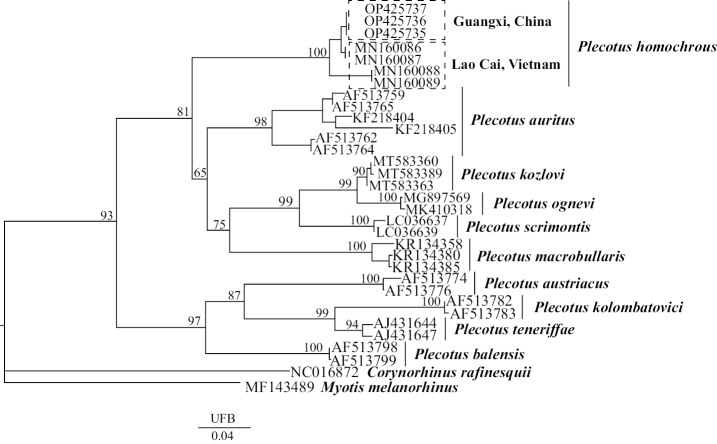
Phylogenetic tree of bats constructed based on results from the maximum-likelihood (ML) analysis of Cyt *b* gene sequences. Numbers on ML tree nodes are ultrafast bootstrap (UFB) support values.

The phylogenetic tree reveals two major clades. The first clade contains *P.auritus*, *P.homochrous*, *P.kozlovi*, *P.macrobullaris*, *P.ognevi*, and *P.sacrimontis* (i.e. *P.auritus* group). The second one includes *P.austriacus*, *P.balensis*, *P.kolombatovici*, and *P.teneriffae* (i.e. the *P.austriacus* group). Bats GD-221656, GD-221657, and GD-221659 are clustered with *P.homochrous* from Vietnam (Fig. 5).

## ﻿Discussion

**Figure 6.**
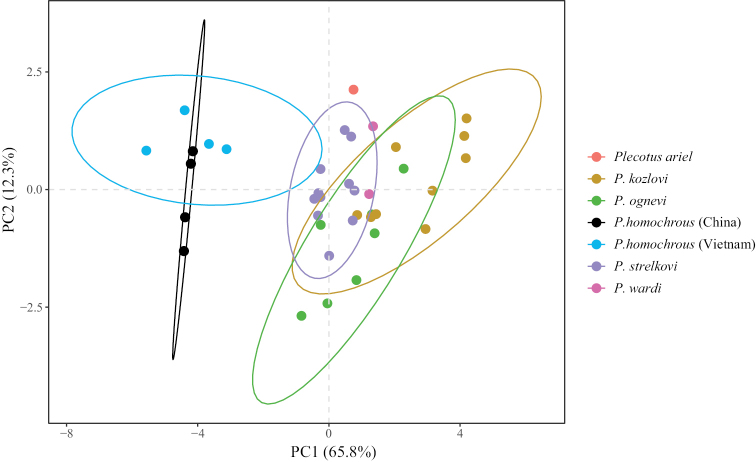
Plots of the first (PC1) versus the second (PC2) principal component for *Plecotusariel*, *P.kozlovi*, *P.ognevi*, *P.homochrous* (examined in this study and those from Vietnam), *P.strelkovi*, and *P.wardi*.

In this study, we identified four bats captured from Guangxi, China as *P.homochrous* based on their morphological characteristics and phylogenetic relationship. In addition to these individuals of *P.homochrous*, bats of six other *Plecotus* species have been found in China, including *P.ariel*, *P.kozlovi*, *P.ognevi*, *P.strelkovi*, *P.taivanus* (Yoshiyuki, 1991), and *P.wardi* (Wilson and Mittermeier 2019; Wei 2022). Among these, *P.homochrous* has the smallest skull and body size and thus are readily distinguishable from the others (Fig. 6). Other major differences include fur color and thumb length. Both ventral and dorsal fur of the four bats are bicolored (ventral fur dark to mixed grey and yellowish; dorsal fur dark to brown), but the fur of other species varies in color pattern as follows: *P.ariel*: ventral, slightly pale; dorsal, grizzled dark brown; *P.kozlovi*: ventral, pale or whitish; *P.ognevi*: ventral, bicolored (with pale brown base and white tips); *P.strelkovi*: dorsal, tricolored (black base, straw-colored middle shaft, and pale tips). Thumb lengths, excluding claws, of the four bats are 3.28–4.46 mm, but those of other bat species are longer (*P.kozlovi*, 7.20–7.60 mm; *P.ognevi*, 7.50–8.30 mm). The major difference between the four *P.homochrous* bats and *P.wardi* is that they have a smaller second upper incisor. Compared to *P.taivanus*, the four bats have a longer forearm (FA) and shorter head body (HB) and tail (T) length than *P.taivanus* [(FA/(HB+T), 41.5% vs 39.0%)]. In addition, the four bats have a keel, but *P.taivanus* lacks such structure.

**Table 4.** List of bat species used in phylogenetic analyses.

Species	Locality	Cyt *b*
*Corynorhinusrafinesquii*	United States	NC016872
*Myotismelanorhinus*	United States	MF143489
*Plecotusauritus*	Guadalajara, Spain	AF513762
	La Rioja, Spain	AF513764
*P.auritus*	Valais, Switzerland	AF513759
	Navarra, Spain	AF513765
	Kırklareli, Turkey	KF218404
	Rize, Turkey	KF218405
*P.austriacus*	Mainz, Germany	AF513774
	Granada, Spain	AF513776
*P.balensis*	Abune Yusef, Ethiopia	AF513798
	Abune Yusef, Ethiopia	AF513799
*P.homochrous*	Guangxi, China	OP425735
	Guangxi, China	OP425736
	Guangxi, China	OP425737
	Lao Cai, Vietnam	MN160086
	Lao Cai, Vietnam	MN160087
	Lao Cai, Vietnam	MN160088
	Lao Cai, Vietnam	MN160089
*P.kolombatovici*	Cyrenaica, Libya	AF513782
	Cyrenaica, Libya	AF513783
*P.kozlovi*	Mongolian	MT583360
	Mongolian	MT583363
	Mongolian	MT583369
*P.macrobullaris*	Italy	KR134358
	Greece	KR134380
	Montenegro	KR134385
*P.ognevi*	Hovsgol National Park, Mongolia	MK410318
	Baikal, Russian	MG897569
*P.sacrimontis*	Oita, Japan	LC036637
	Hokkaido, Japan	LC036639
*P.teneriffae*	La Palma, Spain	AJ431644
	El Hierro, Spain	AJ431647

Although the four bats are morphologically and phylogenetically identical to *P.homochrous* from Vietnam, the pulse duration of their echolocation calls is significantly longer than in *P.homochrous* from Vietnam; such differences may be due to the complexity in recording echolocation calls, as bats tend to send more pulses to obtain sufficient information when they fly in complex environments (Siemers et al. 2001; Dietrich et al. 2006; Peng et al. 2019). The relatively high frequency of maximum energy and low wing loading and wingspan ratio of the bats suggest that they forage in relatively dense and complex environment using gleaning strategy and are montane forest dweller.

Although many *Plecotus* species have been found in China, detailed information on their geographical distribution is not available (Yu et al. 2021), and the identity of two of these species (*P.ariel* and *P.taivanus*) remains uncertain because of the lack of molecular evidence. There are also two *Plecotus* species found in Xizang and Gansu, China that have yet to be named (Fig. 1; Spitzenberger et al. 2006). A well-defined list of species diversity can provide important information for the designation of protected areas for ecological conservation of various bat species. As such list is currently lacking, further efforts to identify novel bat species and investigate their distribution ranges in China are warranted.

## Supplementary materials

Supplementary material 1 Additional information Data type tables (docx. file) Explanation note table S1: references of *Plecotus* species investigated; table S2: Factor loading scores of characteristics used for the PCA of six bat species from China and other regions. https://binary.pensoft.net/file/849009This dataset is made available under the Open Database License (http://opendatacommons.org/licenses/odbl/1.0/). The Open Database License (ODbL) is a license agreement intended to allow users to freely share, modify, and use this Dataset while maintaining this same freedom for others, provided that the original source and author(s) are credited.Pengfei Luo
