# Supplementary material for: ﻿Confirmation of the existence of Himalayan long-eared bats, Plecotushomochrous (Chiroptera, Vespertilionidae), in China
Source: Zookeys. 2023 May 11;1161:129–41. doi: 10.3897/zookeys.1161.99487 (PMC10206652; doi:10.3897/zookeys.1161.99487)
Supplement: Supplementary material 1 — Additional information [file zookeys-1161-129_article-99487__-s001.docx]

Supplementary file **Table S1.** References of *Plecotus* species investigated.

| Species | Number | Locality | Literature obtained |
| --- | --- | --- | --- |
| *Plecotus homochrous* | IEBR-M-5469 | Lao Cai, Vietnam | Dai et al. 2020 |
|  | IEBR-M-5472 | Lao Cai, Vietnam | Dai et al. 2020 |
|  | IEBR-M-5483 | Lao Cai, Vietnam | Dai et al. 2020 |
|  | HNHM202011 | Lao Cai, Vietnam | Dai et al. 2020 |
| *P. ariel* | BMNH 11216 | Sichuan, China | Spitzenberger et al. 2006 |
| *P. wardi* | BMNH 61032 | Near Leh, Kashmir, India | Spitzenberger et al. 2003 |
|  | BMNH 61031 | Near Leh, Kashmir, India | Spitzenberger et al. 2003 |
| *P. ognevi* | 1576 | Mongolia | Dolch et al. 2021 |
|  | 120 | Mongolia | Dolch et al. 2021 |
|  | 786 | Mongolia | Dolch et al. 2021 |
|  | 1260 | Mongolia | Dolch et al. 2021 |
|  | 1262 | Mongolia | Dolch et al. 2021 |
|  | 1377 | Mongolia | Dolch et al. 2021 |
|  | 1428 | Mongolia | Dolch et al. 2021 |
| *P. kozlovi* | 1605 | Mongolia | Dolch et al. 2021 |
|  | 1610 | Mongolia | Dolch et al. 2021 |
|  | 1577 | Mongolia | Dolch et al. 2021 |
|  | 1608 | Mongolia | Dolch et al. 2021 |
|  | 1609 | Mongolia | Dolch et al. 2021 |
|  | 1607 | Mongolia | Dolch et al. 2021 |
|  | 276 | Mongolia | Dolch et al. 2021 |
|  | 843 | Mongolia | Dolch et al. 2021 |
|  | 844 | Mongolia | Dolch et al. 2021 |
| *P. strelkovi* | 1606 | Mongolia | Dolch et al. 2021 |
|  | 1581 | Mongolia | Dolch et al. 2021 |
|  | 1570 | Mongolia | Dolch et al. 2021 |
|  | 1617 | Mongolia | Dolch et al. 2021 |
|  | 1619 | Mongolia | Dolch et al. 2021 |
|  | 1620 | Mongolia | Dolch et al. 2021 |
|  | 1572 | Mongolia | Dolch et al. 2021 |
|  | 1573 | Mongolia | Dolch et al. 2021 |
|  | 1618 | Mongolia | Dolch et al. 2021 |
|  | 1621 | Mongolia | Dolch et al. 2021 |
|  | 1569 | Mongolia | Dolch et al. 2021 |
| *P. taivanus* | NSMT-M 29614 | Taiwan, China | Yoshiyuki 1991 |
|  | NSMT-M 29615 | Taiwan, China | Yoshiyuki 1991 |
|  | NSMT-M 29616 | Taiwan, China | Yoshiyuki 1991 |

**Table S2.** Factor loading scores of characteristics used for the PCA of six bat species from China and other regions.

| Characteristics | PC1 | PC2 |
| --- | --- | --- |
| STOTL | 0.347 | −0.198 |
| CBL | 0.371 | −0.116 |
| MAW | 0.330 | −0.062 |
| CM^3^L | 0.343 | 0.004 |
| M^3^M^3^W | 0.354 | −0.077 |
| CM_3_L | 0.357 | −0.086 |
| BCW | 0.293 | 0.151 |
| BCH | 0.343 | −0.155 |
| Bulla | 0.220 | 0.536 |
| IOW | 0.103 | 0.772 |
| Eigenvalues | 2.565 | 1.110 |
| Percentage of total variance | 65.8 | 12.3 |
| Cumulative percentage | 65.8 | 78.1 |

**References**

Dai F, Tu VT, Thanh HT, Arai S, Harada M, Csorba G, Son NW (2020) First record of the genus *Plecotus* from Southeast Asia with notes on the taxonomy, karyology and echolocation call of *P. homochrous* from Vietnam. Acta Chiropterologica 22: 57–74. <https://doi.org/10.3161/15081109acc2020.22.1.006>

Dolch D, Gärtner B, Thiele K, Steinhause D, Jargalsaikhan A, Stubble A, Batsajchan N, Davaa L (2021) Phylogenie, Morphologie und Ökologie mongolischer Phylogenie, Morphologie und Ökologie mongolischer. Erforschung biologischer Ressourcen der Mongolei 14: 123–185.

Spitzenberger F, Strelkov P, Haring E (2003) Morphology and mitochondrial DNA sequences show that *Plecotus alpinus* Kiefer & Veith, 2002 and *Plecotus microdontus* Spitzenberger, 2002 are synonyms of *Plecotus macrobullaris* Kuzjakin, 1965. Croatian Natural History Museum 12(2): 39–53.

Spitzenberger F, Strelkov P, Winkler H, Haring E (2006) A preliminary revision of the genus *Plecotus* (Chiroptera, Vespertilionidae) based on genetic and morphological results. Zoological Scripta 35: 187–230. <https://doi.org/10.1111/j.1463-6409.2006.00224.x>

Yoshiyuki M (1991) A New species of *Plecotus* (Chiroptera, Vespertilionidae) from Taiwan. Bulletin of the National Science Museum 17(4): 189–195.
